# The Bradykinin System Contributes to the Regulation of Prostaglandin-Endoperoxide Synthase 2 Expression in Human Amnion Fibroblasts: Implications for Term and Preterm Birth

**DOI:** 10.3389/fendo.2022.873727

**Published:** 2022-05-11

**Authors:** Xiao-tian Ni, Wang-sheng Wang, Yun Liu, Yi-kai Lin, Fan Zhang, Wen-jia Lei, Li-jun Ling, Fan Pan, Ya-nan Zhu, Meng-die Li, Tao Duan, Ming Liu, Kang Sun

**Affiliations:** ^1^ Department of Obstetrics and Gynecology, Shanghai East Hospital, Tongji University School of Medicine, Shanghai, China; ^2^ Center for Reproductive Medicine, Ren Ji Hospital, School of Medicine, Shanghai Jiao Tong University, Shanghai, China; ^3^ Shanghai Key Laboratory for Assisted Reproduction and Reproductive Genetics, Shanghai, China; ^4^ Shanghai First Maternity and Infant Hospital, Tongji University School of Medicine, Shanghai, China

**Keywords:** bradykinin, cyclooxygenase-2, PGE2, fetal membranes, preterm birth, parturition, inflammation, chorioamnionitis

## Abstract

**Background:**

Bradykinin (BK) and its biologically active metabolite des-Arg9 bradykinin (DABK) play a pivotal role in inflammation. Since chorioamnionitis is the leading cause of preterm birth and prostaglandin E2 (PGE2) derived from the amnion is key to labor initiation, we investigated if bradykinin peptides are part of the regulatory network of PGE2 synthesis in human amnion at parturition.

**Methods:**

Human amnion tissue was obtained from term and preterm birth for the study of the changes of the bradykinin system at parturition. Cultured primary human amnion fibroblasts, the major source of PGE2, were used to study the effects of bradykinin peptides on PTGS2 expression and PGE2 production as well as the effects of infection mediators on bradykinin receptors.

**Results:**

Bradykinin peptides and their receptors BDKRB1 and BDKRB2 were present in human amnion, and their abundance increased in term and preterm labor. However, transcripts of the genes encoding the bradykinin precursor and its proteolytic cleavage enzymes were hardly detectable in human amnion despite the increased abundance of bradykinin peptides in term and preterm labor, suggesting that there is an alternative source of bradykinin peptides for human amnion and their actions are enhanced in human amnion at parturition. *In-vitro* studies in cultured human amnion fibroblasts showed that both BK and DABK increased the expression of prostaglandin-endoperoxide synthase 2 (PTGS2), the rate-limiting enzyme in prostaglandin synthesis, and subsequent PGE2 production. These effects of BK and DABK were mediated through BDKRB2 and BDKRB1 receptors, respectively, with subsequent activation of the p38 and ERK1/2 pathways. Moreover, lipopolysaccharide (LPS) and serum amyloid A1 (SAA1), the important mediators of infectious inflammation, induced the expression of both BDKRB1 and BDKRB2 through toll-like receptor 4 (TLR4). Induction of BDKRB1 and BDKRB2 expression by LPS and SAA1 enhanced BK- or DABK-induced PTGS2 expression and PGE2 production in human amnion fibroblasts.

**Conclusions:**

This study demonstrated for the first time that the human amnion is a target tissue of bradykinin peptides and the bradykinin system may be part of the regulatory network of PTGS2 expression and PGE2 production in human amnion fibroblasts at both term and preterm birth, which may be enhanced by infection.

## Introduction

**Figure 1:**
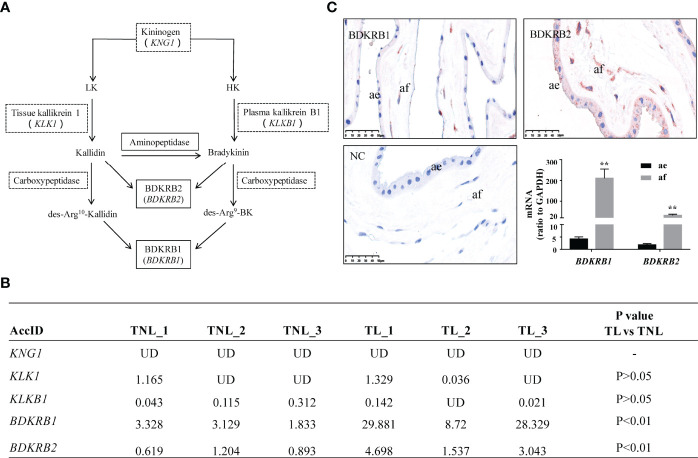
Pathway of bradykinin synthesis and the expression of the bradykinin system in human amnion. **(A)** Pathway of bradykinin synthesis. Italic word in brackets is the gene name of the corresponding protein. UD, undetected. **(B)** FPKM of gene transcripts related to bradykinin synthesis in the amnion at term with labor (TL, *n* = 3) and without labor (TNL, *n* = 3) as revealed by transcriptomic sequencing. **(C)** Immunohistochemical staining of bradykinin B1 receptor (BDKRB1) and bradykinin B2 receptor (BDKRB2) in human amnion, and comparison of *BDKRB1* and *BDKRB2* mRNA expression in human amnion fibroblasts and epithelial cells (*n* = 3). ae, amnion epithelial cells; af, amnion fibroblasts; scale bar, 50 µm. Statistical analysis was performed with unpaired Student’s *t*-test. **p < 0.01 vs. ae.

Bradykinin (BK) is a biologically active nonapeptide formed from the proteolytic cleavage of either high or low molecular weight kininogens (1–3) (**Figure 1A**). The two molecular forms of kininogens are alternatively spliced products of the same gene *KNG1* in humans. The high molecular weight kininogen is converted to bradykinin under the enzymatic action of *KLKB1-*encoded plasma kallikrein, while the low molecular weight kininogen is converted to bradykinin *via* two-step enzymatic reactions. The first step is catalyzed by the *KLK1*-encoded tissue kallikrein which yields Lys-bradykinin, a 10-amino-acid peptide also known as kallidin, and the second step is catalyzed by a plasma aminopeptidase which cleaves the N-terminal Lys residue of kallidin resulting in the formation of bradykinin. Of note, further removal of the Arg residue at the C-terminal of bradykinin by kininase I-type carboxypeptidases produces another biologically active octapeptide [des-Arg9]-BK (DABK) (1, 2, 4).

The effects of BK and DABK are mediated through G-protein-coupled bradykinin receptors. BK is more selective for bradykinin B2 receptor (BDKRB2), while DABK is more specific for bradykinin B1 receptor (BDKRB1). Both BDKRB1 and BDKRB2 are coupled with activation of the PKC/calcium and MAPK pathways and inhibition of the cAMP/PKA pathway (2, 5). It is believed that bradykinin-coupled BDKRB2 is constitutively expressed, whereas DABK-coupled BDKRB1 is inducible under inflammatory conditions (5, 6). Despite the differential expression features of these two bradykinin receptors, activation of either of the receptors has been linked to inflammatory responses (6–8). Of note, excessive accumulation of DABK can even evolve into a bradykinin storm resulting in severe inflammatory responses when its metabolism is left unchecked (9–11), suggesting a crucial role of bradykinin peptides in inflammation. It has been shown that BK and DABK participate in inflammation responses not only by increasing vasodilation and vascular permeability but also by stimulating the production of proinflammatory factors including cytokines and prostaglandins (1, 12–14). In terms of prostaglandin synthesis, bradykinin peptides induce the expression of prostaglandin-endoperoxide synthase 2 (PTGS2), also known as cyclooxygenase-2 (COX-2), the rate-limiting enzyme in prostaglandin synthesis in various cell types (14–19).

Accumulating evidence indicates that parturition is an inflammatory process of the intrauterine tissues, particularly the fetal membranes, decidua, and cervix both in term normal parturition and in infection-induced preterm birth (20–23). In normal term parturition, sterile inflammation of these intrauterine tissues leads to increased production of prostaglandins and proinflammatory cytokines, which, in turn, stimulate myometrial contraction, cervical ripening, and membrane rupture (22–25). When infection is present, inflammation of the intrauterine tissues is exaggerated, which leads to preterm birth, a primary cause of perinatal mortality and morbidity (23). Of the prostaglandins involved in parturition, prostaglandins E2 (PGE2) and F2α (PGF2α) are particularly important. These prostaglandins are believed to be the final common mediators of labor onset in both term and preterm birth as potent inducers of cervical ripening, myometrial contraction, and membrane rupture (26). Virtually, all the intrauterine tissues are, more or less, capable of prostaglandin synthesis in pregnancy, but in humans, the amnion layer of the fetal membranes and the decidua/myometrium are believed to be the major sources of PGE2 and PGF2α, respectively (26, 27). PGE2 synthesized in amnion is a self-inducer of its own synthesis *via* induction of PTGS2 expression thereby reinforcing its own actions in parturition in a feedforward manner (26, 28). In addition to PGE2, a number of other factors including proinflammatory cytokines and glucocorticoids have also been shown to be part of the upregulatory network of PTGS2 expression in amnion at parturition (25, 29–31). However, whether the bradykinin system is also part of this regulatory network in amnion is not known. Elucidation of the role of bradykinin peptides in the regulation of PTGS2 expression in amnion may help further resolve the mechanism of labor onset. In this study, we investigated whether the bradykinin system was present in human amnion and then examined whether there was any change in the abundance of the bradykinin system in amnion in term and preterm birth. Furthermore, we examined the effects of BK and DABK on PTGS2 expression and PGE2 production as well as the mediating receptor and signaling pathway by using cultured primary human amnion fibroblasts, the major source of PGE2 in amnion (32, 33). Because bacterial infection is the most frequent cause of preterm birth, we also examined whether the bacterial product lipopolysaccharide (LPS) and serum amyloid A1 (SAA1), an acute-phase protein produced in large amounts following infection (34), were capable of modulating the effects of BK and DABK on PTGS2 expression in human amnion fibroblasts.

## Materials and Methods

### Collection of Human Fetal Membranes

Human placentae were obtained immediately from deliveries with written informed consent from participating women under a protocol [Protocol No. (2013) N025] approved by the Ethics Committee of Ren Ji Hospital, School of Medicine, Shanghai Jiao Tong University. Modes of deliveries included uncomplicated term pregnancies after elective cesarean section without labor (term non-labor, TNL); spontaneous labor at term (term labor, TL); preterm pregnancies terminated by cesarean section without labor for maternal or fetal conditions including placenta previa, vasa previa, and fetal distress (preterm non-labor, PNL); and spontaneous preterm labor with no indication of infection (preterm labor, PL). Pregnancies with complications such as preeclampsia, fetal growth restriction, and gestational diabetes were excluded. Within 20 min of delivery, the placentae were transported in cold normal saline to the laboratory and the reflected fetal membranes were cut from the placenta, and the amnion layer was peeled off from the chorion layer. For RNA and protein extraction, the amnion layer was cut within 5 cm of the spontaneous or artificial rupture site and rinsed with ice-cold normal saline to remove residual blood and then placed in liquid nitrogen for storage at −80°C until RNA and protein extraction. RNA extracted from the amnion in the TNL and TL groups was processed for transcriptomic sequencing for the initial investigation of the bradykinin system in amnion, which has been reported in a previous publication of our laboratory (28). RNA extracted from the amnion in the TNL, TL, PNL, and PL groups was also processed for measurements with quantitative real-time-polymerase chain reaction (qRT-PCR) to confirm the changes of the bradykinin system in labor observed with transcriptomic sequencing. Protein extracted from the amnion in the TNL, TL, PNL, and PL groups was used for the measurements of bradykinin changes in labor with enzyme-linked immunosorbent assay (ELISA). To investigate the distribution of BDKRB1 and BDKRB2 in human amnion, amnion tissue at the rupture site from TL patients (*n* = 3) was processed for staining with immunohistochemistry. To study the effects of BK, DABK, LPS, and SAA1 on the expression of PTGS2, BDKRB1, or BDKRB2 and the underlying mechanisms, fibroblasts were isolated from the entire amnion of the reflected membranes collected from the cesarean section at term without labor. The number of women from whom the fetal membranes were collected was given in the corresponding figure legend for each study. Demographic and clinical features of pregnant women are given in **Tables 1** and **2**.

**Table 1:** Demographic and clinical characteristics of recruited pregnant women in term non-labor (TNL) and term labor (TL) groups.

Demographic features	TNL	TL	*p*-value
Maternal age (years)	32.1 ± 0.5	30.6 ± 0.6	0.0686
Body mass index (kg/m^2^)	25.2 ± 1.0	24.7 ± 0.5	0.6457
Gravidity [median (range)]	2 (1–5)	1 (1–2)	0.0672
Parity [median (range)]	1 (1–2)	1 (1–2)	0.3707
Gestational age at delivery (weeks)	38.6 ± 0.2	38.9 ± 0.2	0.3566
Birth weight (g)	3,523 ± 69.3	3,198 ± 113.5	0.0931

TNL: n = 11; TL: n = 12. Maternal age, body mass index, gestational age at delivery, and birth weight were expressed as mean ± SD and analyzed with unpaired Student’s t-test. Gravidity and parity were expressed as median (min–max) and analyzed with the Mann–Whitney U test. Significance was set at P < 0.05.

**Table 2:** Demographic and clinical characteristics of recruited pregnant women in preterm non-labor (PNL) and preterm labor (PL) groups.

Demographic features	PNL	PL	*p*-value
Maternal age (years)	34.3 ± 2.2	30.7 ± 1.1	0.1301
Body mass index (kg/m^2^)	25.2 ± 0.9	24.8 ± 0.9	0.7695
Gravidity [median (range)]	2 (1–4)	1 (1–5)	0.1991
Parity [median (range)]	1 (1–2)	1 (1–1)	0.0515
Gestational age at delivery (weeks)	34.3 ± 0.4	33.6 ± 0.5	0.5425
Birth weight (g)	2,356.0 ± 89.5	2,134.0 ± 112.1	0.1695

PNL: n = 7; PL: n = 11. Maternal age, body mass index, gestational age at delivery, and birth weight were expressed as mean ± SD and analyzed with unpaired Student’s t-test. Gravidity and parity were expressed as median (min–max) and analyzed using Mann–Whitney U test. Significance was set at P < 0.05.

### Measurements of Bradykinin Peptides in Human Amnion

To compare the amounts of bradykinin peptides in amnion with or without labor at term and preterm, amnion tissue pieces were cut 5 cm within the rupture site of the amnion and then ground in liquid nitrogen. The ground tissue was homogenized and lysed in ice-cold RIPA lysis buffer containing a protease inhibitor cocktail and centrifuged. Protein in the supernatant was collected for bradykinin peptide measurement with an ELISA kit (Abcam, Cambridge, MA, USA, #ab136936) with no distinction between BK and DABK, following the protocol provided by the manufacturer.

### Immunohistochemical Staining of BDKRB1 and BDKRB2 in Human Amnion

Tissue sections were cut from paraffin-embedded human amnion tissue from TL after fixing in 4% paraformaldehyde. After quenching the endogenous peroxidase activity with 0.3% H_2_O_2_ and blocking with normal serum, primary antibodies against human BDKRB1 (Proteintech, Wuhan, China, #26672-1-AP) and BDKRB2 (R&D System, Minneapolis, MN, USA, #MAB9434-SP) were applied to the section at 1:200 dilutions for overnight incubation at 4°C. After washing with PBS, a secondary antibody conjugated with biotinylated horseradish peroxidase was applied to the section for further incubation for 1 h at room temperature. The red color was then developed using the substrate 3-amino-9-ethyl carbazole (Vector Laboratories, Burlingame, CA, USA). The slide was counterstained with hematoxylin (blue color) and examined under a regular light microscope (Zeiss, Oberkochen, Germany).

### Isolation and Culture of Human Amnion Epithelial Cells and Fibroblasts

Amnion fibroblasts were isolated from the amnion collected from TNL as described previously (35). Briefly, the amnion tissue was digested twice with 0.125% trypsin (Sigma Chemical Co., St. Louis, MO, USA) and then washed thoroughly with normal saline to remove epithelial cells for culture. The remaining tissue was digested with 0.1% collagenase (Sigma) to isolate fibroblasts, and fibroblast cells in the digestion medium were collected by centrifugation. Isolated epithelial cells and fibroblasts were cultured at 37°C in 5% CO_2_–95% air in DMEM containing 10% fetal calf serum (FCS) plus 1% antibiotics (all from Life Technologies Inc., Grand Island, NY, USA) for 3 days before treatments. The identity of cells was verified by staining for mesenchymal cell marker vimentin. More than 95% of the cells were positive for vimentin in amnion fibroblast preparation and the epithelial cells were pure and negative for vimentin.

### Treatment of Human Amnion Fibroblasts

Three days after plating, the culture medium was replaced with DMEM free of phenol red and FCS for treatments of amnion fibroblasts. To investigate the effects of BK and DABK on the expression of PTGS2, time course and concentration-dependent studies were conducted. For the time course study, fibroblasts were treated with BK (0.1 µM, Tocris, Minneapolis, MN, USA) or DABK (0.1 µM, MedChemExpress, Princeton, NJ, USA) for 1, 2, 4, 8, and 12 h. For the concentration-dependent study, fibroblasts were treated with BK or DABK for 4 h at concentrations of 0.01, 0.1, and 1 µM. To examine the receptor subtype involved, fibroblasts were treated with BK (0.1 µM) or DABK (0.1 µM) for 4 h in the presence or absence of the BDKRB1 antagonist ELN-441958 (1 µM, MedChemExpress) or the BDKRB2 antagonist icatibant (1 µM, MedChemExpress). The antagonists were added 1 h before BK or DABK treatment. To examine the downstream signaling pathway involved in the induction of PTGS2 expression by BK or DABK, a time course study (15, 30, 60, 120, and 180 min) was carried out to examine the effect of BK (0.1 µM) or DABK (0.1 µM) on the phosphorylation of MAPK members including p38, ERK1/2, and JNK. To further investigate the involvement of MAPK members in the induction of PTGS2 expression by BK or DABK, fibroblasts were treated with BK (0.1 µM) or DABK (0.1 µM) for 4 h in the presence or absence of the p38 inhibitor SB203580 (10 µM; Selleck, Houston, TX, USA), the ERK1/2 inhibitor PD98059 (20 µM; Selleck), or the JNK inhibitor SP600125 (10 µM; Sigma). The inhibitors were added 1 h before BK or DABK treatment.

To study the effects of SAA1 and LPS on BDKRB1 and BDKRB2 expression in amnion fibroblasts, cells were treated with recombinant human apo-SAA1 (10, 50, and 100 ng/ml; PeproTech Inc., Rocky Hill, NJ, USA) or LPS (1, 10, and 50 ng/ml; Sigma) for 24 h. The involvement of toll-like receptor 4 (TLR4) in the effects of SAA1 and LPS on BDKRB1 and BDKRB2 was studied by treating the cells with SAA1 (50 ng/ml) or LPS (50 ng/ml) in the presence or absence of the TLR4 receptor antagonist CLI-095 (5 µM, Invitrogen, San Diego, CA, USA) for 24 h. To examine the effect of SAA1 and LPS on the induction of PTGS2 expression by BK and DABK, the cells were pretreated with SAA1 (50 ng/ml) or LPS (50 ng/ml) for 24 h, and then the culture medium was replaced with medium containing only BK (0.1 µM) or DABK (0.1 µM) for further incubation for 4 h.

### Measurements of *BDKRB1*, *BDKRB2*, and *PTGS2* mRNA Abundance With qRT-PCR

For the measurement of *BDKRB1* and *BDKRB2* mRNA abundance in human amnion tissue, the snap-frozen tissue was ground in liquid nitrogen and homogenized. Total RNA was extracted from the homogenized tissue with a total RNA isolation Kit (Foregene, Chengdu, China). For the measurement of *BDKRB1*, *BDKRB2*, and *PTGS2* mRNA abundance in human amnion fibroblasts or epithelial cells, total RNA was extracted from the cells with the same total RNA isolation Kit. The mRNA in total RNA was reverse-transcribed to cDNA using a commercial kit (TaKaRa, Tokyo, Japan). The reverse-transcribed cDNA was used to measure *BDKRB1*, *BDKRB2*, and *PTGS2* mRNA in power SYBR^®^ Premix Ex Taq™ (TaKaRa) with qRT-PCR. The housekeeping gene glyceraldehyde 3-phosphate dehydrogenase (*GAPDH*) was amplified in parallel as an internal control. The abundance of mRNA was quantified using the 2−^△△Ct^ method. The primer sequences used for qRT-PCR are illustrated in **Table 3**.

**Table 3:** Primer sequences used for qRT-PCR.

Genes	Primer sequences
*BDKRB1*	Forward: 5′-GCAACTGAACGTGGCAGAAA-3′
	Reverse: 5′-GCAAGCCCAAGACAAACACC-3′
*BDKRB2*	Forward: 5′-CAACCCACTGGTGTACGTGA-3′
	Reverse: 5′-CAGTGTGCCCATGGAGTTCT-3′
*PTGS2*	Forward: 5′-TGTGCAACACTTGAGTGGCT-3′
	Reverse: 5′-ACTTTCTGTACTGCGGGTG-3′
*GAPDH*	Forward: 5′-CCCCTCTGCTGATGCCCCCA-3′
	Reverse: 5′-TGACCTTGGCCAGGGGTGCT-3′

### Measurements of PTGS2, BDKRB1, and BDKRB2 and MAPK Protein Abundance With Western Blotting

After treatments, human amnion fibroblasts were lysed in ice-cold radioimmunoprecipitation assay (RIPA) buffer (Active Motif, Carlsbad, CA, USA) containing a protease inhibitor cocktail (Roche, Basel, Switzerland) and a phosphatase inhibitor (Roche) for the extraction of total cell protein for the measurements of PTGS2, MAPKs, and phosphorylated MAPKs with Western blotting. For the measurements of membrane BDKRB1 and BDKRB2 protein abundance, membrane protein was extracted with a differential centrifugation method using a commercial kit (Beyotime, Shanghai, China). After determination of protein concentration, the extracted protein was analyzed with Western blotting following a standard protocol. The primary antibodies used for Western blotting were as follows: p38 (1:500, Cell Signaling, Danvers, MA, USA, #8690), phosphorylated p38 at Thr180/Tyr182 (1:500, Cell Signaling, #4511), ERK1/2 (1:500, Cell Signaling, #4631), phosphorylated ERK1/2 at Thr202/Tyr204 (1:500, Cell Signaling, #4370), JNK (1:1,000, Cell Signaling, #9252), phosphorylated JNK at Thr183/Tyr185 (1:1,000, Cell Signaling, #9255), BDKRB1 (1:100, Santa Cruz, Santa Cruz, CA, USA, #sc-293196), BDKRB2 (1:500, Alomone, Jerusalem BioPark, Israel, #ABR-012), and PTGS2 (1:1,000, Cell Signaling, #12282). After incubation with primary antibodies for 24 h, the corresponding secondary antibodies conjugated with horseradish peroxidase were applied to further incubate for 1 h. Peroxidase activity was developed by a chemiluminescence detection system (Millipore, Billerica, MA, USA) and visualized using a G-Box chemiluminescence image capture system (Syngene, Cambridge, UK). Internal loading controls were probed with a GAPDH antibody (1:10,000, Proteintech, #60004-1-Ig) for total cell protein or Na^+^/K^+^ ATPase antibody (1:500, Cell Signaling, #23565) for membrane protein.

### Measurements of PGE2 in the Culture Medium of Human Amnion Fibroblasts

After treatment with BK (0.1 µM) or DABK (0.1 µM) for 4 h or pretreatment with SAA1 (50 ng/ml, 24 h) and LPS (50 ng/ml, 24 h) followed by BK (0.1 µM) or DABK (0.1 µM) treatment for 4 h, PGE2 in the culture media was measured with an enzyme immunoassay kit (Cayman, Ann Arbor, MI, USA) according to the protocol provided by the manufacturer.

### Statistical Analysis

All data are reported as means ± SEM. The number of each study represents separate experiments using amnion from different women. The Shapiro–Wilk normality test was used to examine the normal distribution of the data. Unpaired Student’s *t*-test was employed for unpaired data of two groups with normal distribution. The Wilcoxon signed-rank test and Mann–Whitney *U* test were used for paired data and unpaired data of two groups, respectively, when the data were not normally distributed. One-way ANOVA test followed by Tukey test or Dunnett’s test was performed to analyze paired data of more than two groups with normal distribution. For the analysis of SAA1 or LPS interaction with BK or DABK, the two-way ANOVA test was employed. Significance was set at *p <*0.05.

## Results

### Examination of the Bradykinin System in Human Amnion

The transcriptomic sequencing data revealed that the transcripts of *KNG1*, *KLKB1*, and *KLK1*, the genes encoding kininogen, plasma, and tissue kallikrein, respectively, were hardly detectable in human amnion, suggesting that bradykinin peptides cannot be synthesized locally in human amnion. By contrast, *BDKRB1* and *BDKRB2* transcripts, the genes encoding BDKRB1 and BDKRB2, respectively, were detected in human amnion with more abundant *BDKRB1* transcripts than *BDKRB2* transcripts (**Figure 1B**). Moreover, the transcriptomic sequencing data showed that the abundance of both *BDKRB1* and *BDKRB2* transcripts was significantly increased in human amnion at TL as compared with TNL (**Figure 1B**). The transcriptomic sequencing data are available at NCBI GEO with the accession number GSE166453. Immunohistochemical staining showed that both BDKRB1 and BDKRB2 were distributed mainly in mesenchymal fibroblasts of human amnion although BDKRB2 appeared also in amnion epithelial cells (**Figure 1C**), which was confirmed by measuring *BDKRB1* and *BDKRB2* mRNA in amnion fibroblasts and epithelial cells with qRT-PCR (**Figure 1C**). These data suggest that amnion mesenchymal fibroblasts are an important target of bradykinin actions which may be enhanced at parturition.

**Figure 2:**
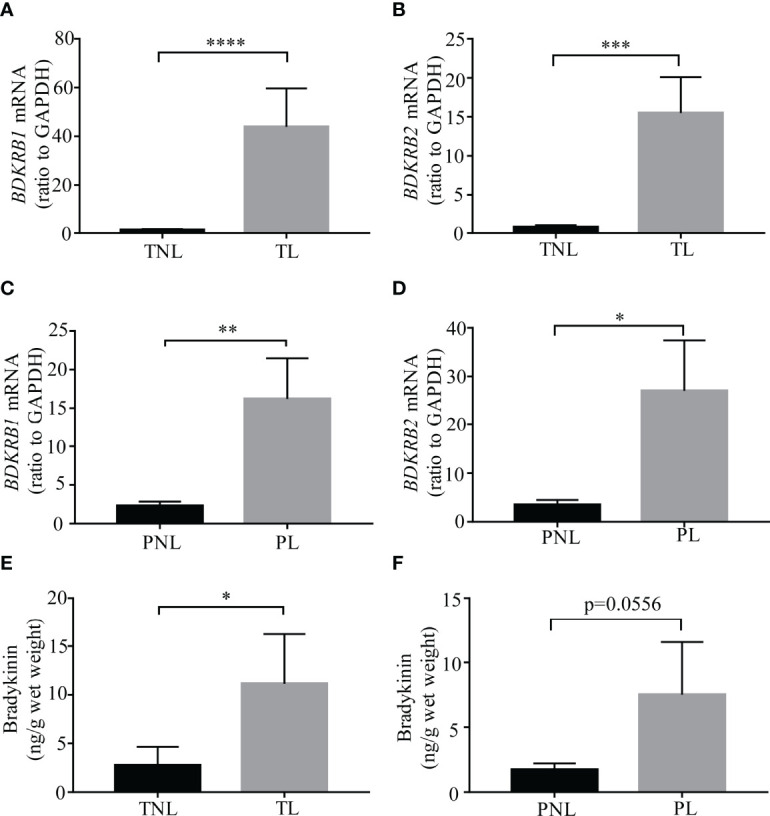
Bradykinin and its receptor abundance in human amnion at term and preterm birth with labor. **(A, B)** *BDKRB1* and *BDKRB2* mRNA abundance in the amnion of term labor (TL, *n* = 12) and term non-labor (TNL, *n* = 11) groups. **(C, D)** *BDKRB1* and *BDKRB2* mRNA abundance in the amnion of preterm labor (PL, *n* = 11) and preterm non-labor (PNL, *n* = 7). **(E)** Bradykinin abundance in the amnion of TL (*n* = 14) and TNL (*n* = 13) groups. **(F)** Bradykinin abundance in the amnion of PL (*n* = 11) and PNL (*n* = 7) groups. Statistical analysis was performed with the Mann–Whitney *U* test. *p < 0.05, **p < 0.01, ***p < 0.001, ****p < 0.0001 vs. TNL or PNL.

Assay with qRT-PCR confirmed the increases in *BDKRB1* and *BDKRB2* mRNA abundance in human amnion at TL (**Figures 2A, B**). In addition, increases in *BDKRB1* and *BDKRB2* mRNA abundance were also observed in human amnion at PL (**Figures 2C, D**). Despite the absence of local bradykinin synthesis, bradykinin peptides were detected with ELISA in human amnion, which was also increased at both TL and PL (**Figures 2E, F**). These data suggest that bradykinin peptides detected in human amnion may be derived from adjacent tissues or maternal blood, and the actions of bradykinin peptides are enhanced in human amnion at both term and preterm labor.

### Effects of BK and DABK on PTGS2 Expression in Human Amnion Fibroblasts

**Figure 3:**
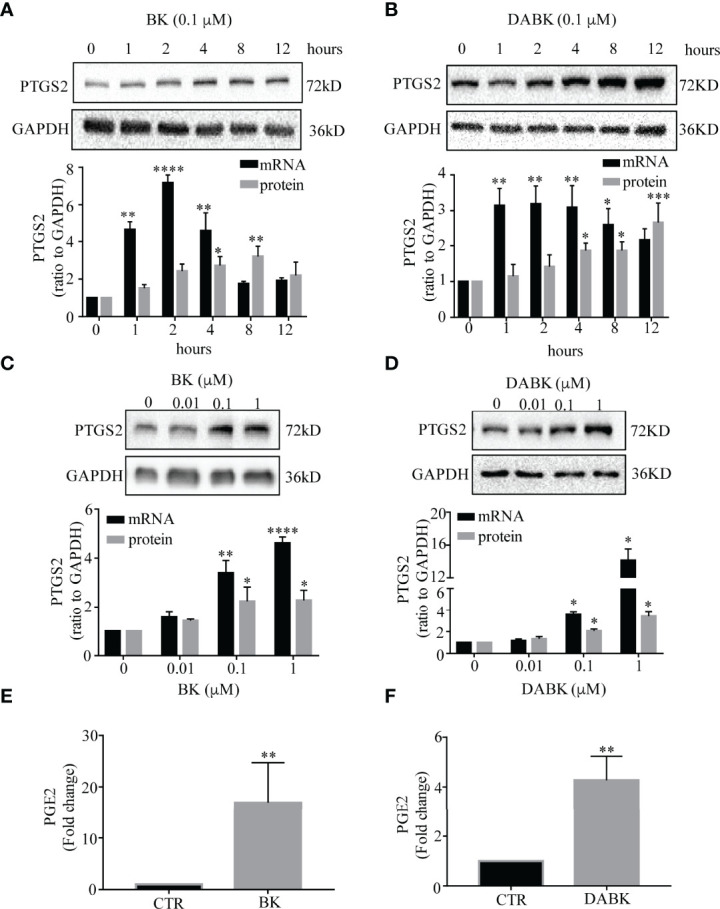
Effects of bradykinin (BK) and des-Arg9 bradykinin (DABK) on PTGS2 expression and PGE2 production in human amnion fibroblasts. **(A)** Time-dependent induction of PTGS2 mRNA (*n* = 3) and protein (*n* = 5) expression by BK (0.1 μM). **(B)** Time-dependent induction of PTGS2 mRNA (*n* = 4) and protein (*n* = 4) expression by DABK (0.1 μM). **(C)** Concentration-dependent induction of PTGS2 mRNA (*n* = 4) and protein (*n* = 4) expression by BK (0, 0.01, 0.1, 1 μM, 4 h). **(D)** Concentration-dependent induction of PTGS2 mRNA (*n* = 3), protein (*n* = 4) expression by DABK (0, 0.01, 0.1, 1 μM, 4 h). Top panels are the representative immunoblots. Statistical analysis was performed with one-way ANOVA test followed by Dunnett’s test. *p < 0.05, **p < 0.01, ***p < 0.001, ****p < 0.0001 vs. 0 h or 0 μM. **(E, F)** Induction of PGE2 production by BK (0.1 μM, 4 h) (*n* = 9) and DABK (0.1 μM, 4 h) (*n* = 10). Statistical analysis was performed with Wilcoxon signed-rank test. **p < 0.01 vs. control (CTR).

Time course studies showed that both BK and DABK induced PTGS2 expression in human amnion fibroblasts in a time-dependent manner (**Figures 3A, B**). Significant increases in PTGS2 mRNA were observed with BK (0.1 μM) treatment for 1, 2, and 4 h but not for 8 and 12 h with maximal effect observed around 2 h. DABK (0.1 μM) treatment significantly increased PTGS2 mRNA abundance in amnion fibroblasts at 1, 2, 4, and 8 h with maximal effect observed between 1 and 4 h. The changes in PTGS2 protein lagged behind that of mRNA. Significant increases in PTGS2 protein were observed at 4 and 8 h after BK (0.1 μM) treatment and at 4, 8, and 12 h after DABK (0.1 μM) treatment. Since both PTGS2 mRNA and protein were significantly increased at 4 h, subsequent studies adopted the incubation with BK and DABK for 4 h.

The induction of PTGS2 mRNA and protein by BK and DABK in amnion fibroblasts was also concentration-dependent (**Figures 3C, D**). Significant increases in PTGS2 mRNA and protein abundance were observed at concentrations greater than 0.1 μM for both BK and DABK. Therefore, 0.1 μM of BK and DABK was chosen for the subsequent studies. At 0.1 μM, BK and DABK also significantly increased PGE2 concentration in the culture medium of amnion fibroblasts (**Figures 3E, F**).

### The Role of BDKRB1, BDKRB2, and MAPKs in the Induction of PTGS2 Expression by BK and DABK in Human Amnion Fibroblasts

**Figure 4:**
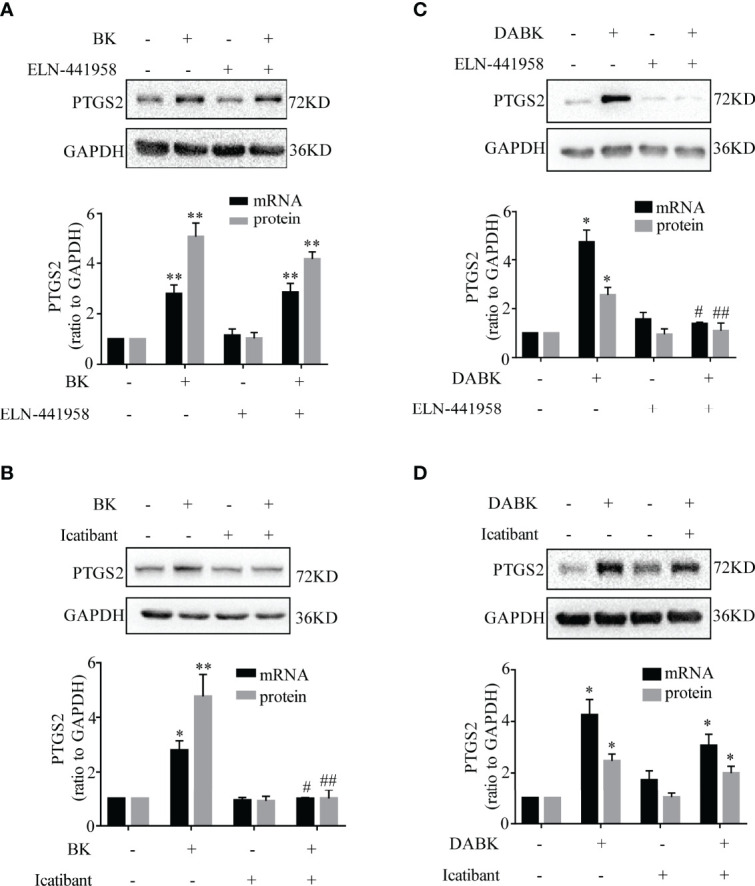
Induction of PTGS2 expression by BK and DABK was mediated by BDKRB2 and BDKRB1, respectively, in human amnion fibroblasts. **(A)** BDKRB1 inhibitor ELN-441958 failed to block BK (0.1 μM, 4 h)-induced PTGS2 mRNA (*n* = 4) and protein (*n* = 3) expression. **(B)** The BDKRB2 inhibitor icatibant blocked BK (0.1 μM, 4 h)-induced PTGS2 mRNA (*n* = 4) and protein (*n* = 3) expression. **(C)** The BDKRB1 inhibitor ELN-441958 blocked DABK (0.1 μM, 4 h)-induced PTGS2 mRNA (*n* = 4) and protein (*n* = 5) expression. **(D)** The BDKRB2 inhibitor icatibant failed to block DABK (0.1 μM, 4 h)-induced PTGS2 mRNA (*n* = 5) and protein (*n* = 6) expression. Statistical analysis was performed with one-way ANOVA test followed by Tukey test. Top panels are the representative immunoblots. *p < 0.05, **p < 0.01 vs. group without BK/DABK and antagonist treatment. ^#^ *p* < 0.05, ^##^ *p* < 0.01 vs. BK- or DABK-treated groups.

**Figure 5:**
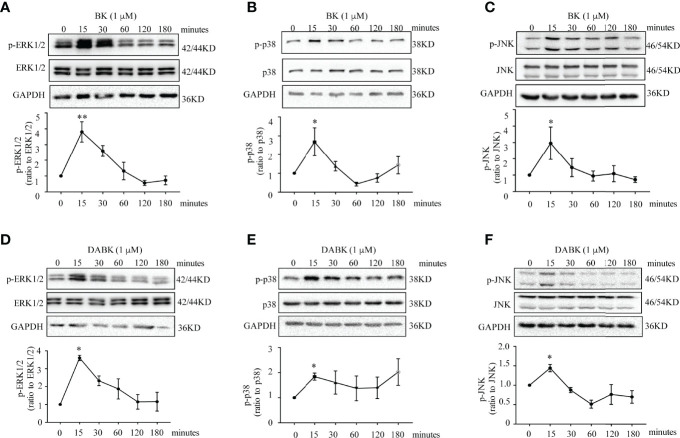
Phosphorylation of p38, ERK1/2, and JNK by BK and DABK in human amnion fibroblasts. **(A–C)** Time-dependent effects of BK (0.1 μM) on p38, ERK1/2, and JNK phosphorylation. **(D–F)** Time-dependent effects of DABK (0.1 μM) on p38, ERK1/2, and JNK phosphorylation. *n* = 3–4. Statistical analysis was performed with one-way ANOVA test followed by Dunnett’s test. Top panels are the representative immunoblots. *p < 0.05, **p < 0.01 vs. 0 min.

**Figure 6:**
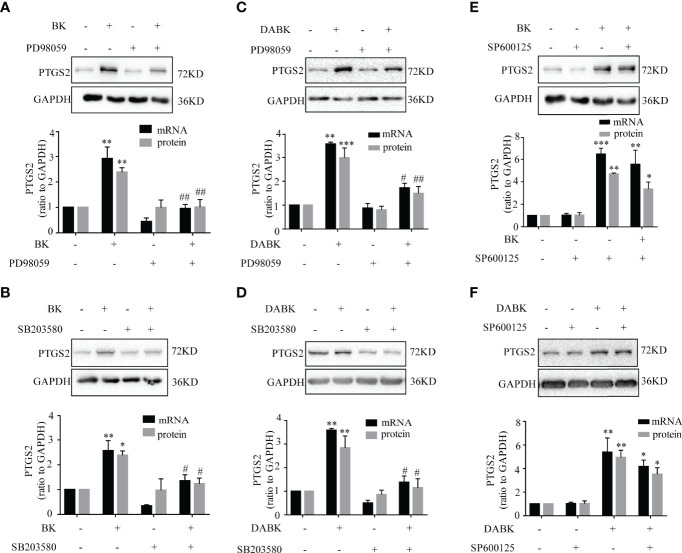
Role of ERK1/2, p38, and JNK in BK- and DABK-induced PTGS2 expression in human amnion fibroblasts. **(A, B)** BK (0.1 μM, 4 h)-induced PTGS2 expression was blocked by the ERK1/2 inhibitor PD98059 (20 µM) (mRNA: *n* = 4; protein: *n* = 3) or the p38MAPK inhibitor SB203580 (10 µM) (mRNA: *n* = 4; protein: *n* = 3). **(C, D)** DABK (0.1 μM, 4 h)-induced PTGS2 expression was blocked by the ERK1/2 inhibitor PD98059 (20 µM) (mRNA: *n* = 3; protein: *n* = 4) or the p38MAPK inhibitor SB203580 (10 µM) (mRNA: *n* = 3; protein: *n* = 4). **(E, F)** The JNK inhibitor SP600125 (10 µM) failed to block the induction of PTGS2 expression by BK (0.1 μM, 4 h) (mRNA: *n* = 4; protein: *n* = 3) or by DABK (0.1 μM, 4 h) (mRNA: *n* = 5; protein: *n* = 3). Statistical analysis was performed with one-way ANOVA test followed by Tukey test. Top panels are the representative immunoblots. *p < 0.05, **p < 0.01, ***p < 0.001 vs. the group without BK/DABK and antagonist. ^#^ *p* < 0.05, ^##^ *p* < 0.01 vs. BK- or DABK-treated groups.

The induction of PTGS2 mRNA and protein by BK (0.1 μM, 4 h) in amnion fibroblasts was blocked by the BDKRB2 antagonist icatibant (1 μM) but not by the BDKRB1 antagonist ELN-441958 (1 μM) (**Figures 4A, B**). By contrast, the induction of PTGS2 expression by DABK (0.1 μM, 4 h) was blocked by the BDKRB1 antagonist ELN-441958 (1 μM) but not by the BDKRB2 antagonist icatibant (1 μM) (**Figures 4C, D**). Both BK (0.1 μM) (**Figures 5A–C**) and DABK (0.1 μM) (**Figures 5D–F**) increased ERK1/2, p38, and JNK phosphorylation significantly in amnion fibroblasts at 15 min after treatments. Inhibition of ERK1/2 and p38 with PD98059 (20 μM) and SB203580 (10 μM), respectively, attenuated the induction of PTGS2 mRNA and protein by BK (0.1 μM) (**Figures 6A, B**) or DABK (0.1 μM) (**Figures 6C, D**) significantly. However, inhibition of JNK with SP600125 (10 µM) failed to block the induction of PTGS2 mRNA and protein by either BK (0.1 μM) or DABK (0.1 μM) (**Figures 6E, F**).

### Induction of BDKRB1 and BDKRB2 Expression by LPS and SAA1 in Human Amnion Fibroblasts

**Figure 7:**
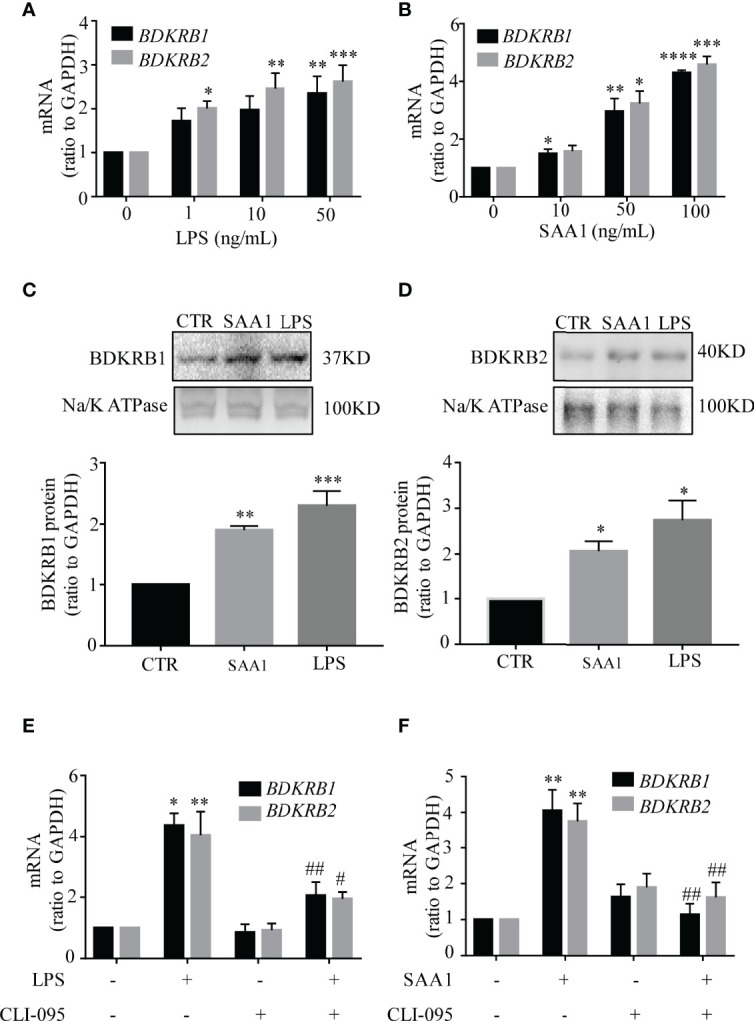
Induction of BDKRB1 and BDKRB2 expression by lipopolysaccharide (LPS) and serum amyloid A1 (SAA1) in human amnion fibroblasts. **(A, B)** Concentration-dependent induction of *BDKRB1* and *BDKRB2* mRNA expression by LPS (0, 1, 10, 50 ng/ml, 24 h) (*n* = 4) and SAA1 (0, 10, 50, 100 ng/ml, 24 h) (*n* = 5). **(C, D)** Upregulation of BDKRB1 and BDKRB2 protein levels by LPS (50 ng/ml, 24 h) and SAA1 (50 ng/ml, 24 h) (*n* = 5). **(E, F)** The TRL4 inhibitor CLI-095 (5 µM) blocked LPS (*n* = 3)- and SAA1 (*n* = 4)-induced *BDKRB1* and *BDKRB2* mRNA expression. Data are the means ± SEM. Statistical analysis was performed with one-way ANOVA test followed by Tukey test. Top panels are the representative immunoblots. *p < 0.05, **p < 0.01, ***p < 0.001, ****p < 0.0001 vs. 0 ng/ml for **(A)** and **(B)**; vs. control (CTR) for **(C)** and **(D)**; vs. group without LPS/SAA1 and CLI-095 treatment for **(E)** and **(F)** ^#^ *p* < 0.05, ^##^ *p* < 0.01 vs. LPS- or SAA1-treated groups.

Both LPS and SAA1 increased *BDKRB1* and *BDKRB2* mRNA abundance in a concentration-dependent manner in amnion fibroblasts. LPS (24 h) significantly increased *BDKRB1* mRNA at 50 ng/ml and increased *BDKRB2* mRNA at 1, 10, and 50 ng/ml (**Figure 7A**). SAA1 (24 h) significantly increased *BDKRB1* mRNA at 10, 50, and 100 ng/ml and *BDKRB2* mRNA at 50 and 100 ng/ml (**Figure 7B**). Consistently, the protein abundance of BDKRB1 and BDKRB2 was also significantly increased by LPS (50 ng/ml, 24 h) and SAA1 (50 ng/ml, 24 h) (**Figures 7C, D**). The induction of *BDKRB1* and *BDKRB2* mRNA by LPS (50 ng/ml, 24 h) (**Figure 7E**) and SAA1 (50 ng/ml, 24 h) (**Figure 7F**) was blocked by the TLR4 antagonist CLI-095 (5 μM).

### Enhancement of BK- and DABK-Induced PTGS2 Expression and PGE2 Production by LPS and SAA1 in Human Amnion Fibroblasts

**Figure 8:**
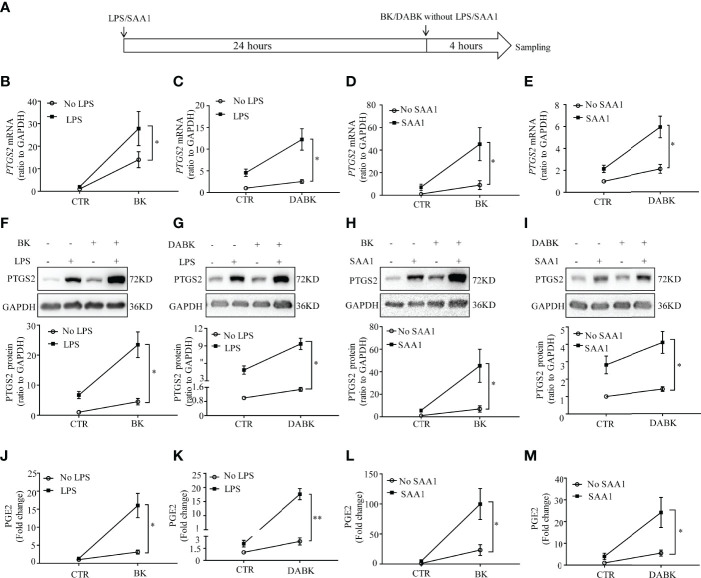
Pretreatment with LPS and SAA1 enhanced the subsequent induction of PTGS2 expression and PGE2 production by BK and DABK in human amnion fibroblasts. **(A)** Time line illustrating pretreatment of amnion fibroblast with LPS (50 ng/ml) or SAA1 (50 ng/ml) for 24 h, followed by BK (0.1 μM) or DABK (0.1 μM) treatment without LPS or SAA1 for another 4 h. **(B–E)** Enhancement of BK (0.1 μM)- and DABK (0.1 μM)-induced *PTGS2* mRNA expression by LPS (50 ng/ml) or SAA1 (50 ng/ml) (*n* = 6). **(F–I)** Enhancement of BK (0.1 μM)- and DABK (0.1 μM)-induced PTGS2 protein expression by LPS (50 ng/ml) or SAA1 (50 ng/ml). *n* = 6 **(F)**; *n* = 5 **(G)**; *n* = 6 **(H)**; *n* = 6 **(I)**. **(J–M)** Enhancement of BK- and DABK (0.1 μM)-induced PGE2 production by LPS (50 ng/ml) or SAA1 (50 ng/ml). *n* = 4 **(J)**; *n* = 4 **(K)**; *n* = 7 **(L)**; *n* = 6 **(M)**. Statistical analysis was performed with two-way ANOVA test. Top panels are the representative immunoblots. *p < 0.05, **p < 0.01 indicate that the interaction factor was significant between groups with and without LPS/SAA1.

Pretreatment of amnion fibroblasts with LPS (50 ng/ml) or SAA1 (50 ng/ml) for 24 h significantly enhanced the subsequent induction of *PTGS2* mRNA (**Figures 8A–E**) and protein expression (**Figures 8F–I**) and PGE2 production (**Figures 8J–M**) by BK (0.1 μM, 4 h) or DABK (0.1 μM, 4 h).

## Discussion

The present study provided evidence for the first time that human amnion was the target tissue for both BK and DABK. We demonstrated that the bradykinin system was part of the upregulatory network of PTGS2 expression and PGE2 production in human amnion at parturition. Given the increased abundance of bradykinin, BDKRB1, and BDKRB2 in human amnion at both term and preterm labor and the pivotal role of PGE2 in labor onset, we propose that the bradykinin system in human amnion is among the driving forces for labor initiation at both term and preterm birth and induction of PTGS2 expression and PGE2 production attributes, at least in part, to the role of the bradykinin system in labor onset. Because both LPS and SAA1 induced the expression of bradykinin receptors and enhanced the induction of PTGS2 expression and PGE2 production by BK and DABK in amnion fibroblasts, we believe that the bradykinin system is also implicated in infection-induced preterm birth given the important role of LPS and SAA1 in infection-induced inflammation (25, 34).

Although both bradykinin and its receptors BDKRB1/BDKRB2 were detected, we failed to detect *KNG1*, *KLKB1*, and *CPN1* transcript*s* in human amnion, which suggests that the human amnion is the target tissue of bradykinin peptides but not their source tissue in pregnancy. It is likely that bradykinin peptides detected in human amnion are derived from neighboring tissues or maternal blood. It is known that circulating kininogen, the precursor of bradykinin, is produced primarily by the liver, although a number of other organs such as the kidney, lung, and heart are also capable of kininogen synthesis (6, 36, 37). However, few studies have addressed the changes of kininogen and bradykinin in maternal blood in pregnancy. An early study by Maki et al. demonstrated that kininogen increased significantly in maternal blood at the end of pregnancy and the beginning of labor, but its level fell at the second stage of labor (38), suggesting that bradykinin formation was increased during labor (39). These findings led us to speculate that increased bradykinin abundance in the amnion in labor may be secondary to the changes in maternal blood.

Because the molecular structures of BK and DABK differ by only one single arginine residue at its carboxyl-terminal, the available commercial kit for BK measurement is not able to distinguish between BK and DABK. Thus, the observed increase in bradykinin abundance in this study may include both BK and DABK. As described in the *Introduction*, the effects of BK and DABK are exerted *via* two specific bradykinin receptors (5, 8). DABK is considered more selective for BDKRB1, while BDKRB2 shows high affinity for BK (5, 8). The findings that BDKRB1 was more abundant than BDKRB2 in human amnion with transcriptomic sequencing are indirect evidence for the presence of both BK and DABK in human amnion.

The bradykinin system is implicated in inflammatory responses through induction of not only vasodilation and fluid expansion but also the production of inflammatory mediators such as cytokines and prostaglandins (7–9, 14, 40). The bradykinin system has been implicated in a number of inflammatory events of female reproduction, including embryo implantation and ovulation (41–43). In addition, the bradykinin system has also been demonstrated to have a role in maintaining uteroplacental blood flow in established gestation as well as in stimulating uterine smooth muscle contraction at parturition (44, 45). In the context of parturition, bradykinin may also augment prostaglandin production through stimulating arachidonic acid release in decidua cells (46). In the present study, we provided evidence that the bradykinin system is part of the driving forces for PGE2 production *via* induction of PTGS2 expression in human amnion fibroblasts in parturition. Given the crucial role of the bradykinin system in inflammatory reactions (10, 12), we believe that the actions of bradykinin may not be restricted only to the induction of PTGS2/PGE2 in parturition. This notion is supported by the findings that bradykinin stimulates interleukin-6 and interleukin-8 secretion in human decidua-derived cells (47). Extracellular matrix remodeling is key to both membrane rupture and cervical ripening in parturition. Interestingly, the bradykinin system has been reported to participate in extracellular matrix remodeling in non-reproductive tissues (8). Thus, it is logical to propose that bradykinin may also be implicated in membrane rupture and cervical ripening through extracellular matrix remodeling in parturition. The effects of bradykinin on proinflammatory cytokines and extracellular remodeling in intrauterine tissues are certainly interesting issues to explore in the future.

Both BDKRB1 and BDKRB2 are coupled through Gqα to stimulate phospholipase C, which results in phosphoinositide hydrolysis, diacylglycerol production, and mobilization of intracellular calcium (2, 5). BDKRB1 and BDKRB2 are also known to act through Giα to inhibit adenylate cyclase (2, 5). Multiple second messengers activated by bradykinin receptors can converge to activate the MAPK pathway (2). The MAPK pathway is a very important signal transduction cascade and a component of a series of vital signal transduction pathways such as cell proliferation, cell differentiation, and cell death (48). The MAPK pathway is also key to the immune response to infection (49). In mammals, MAPK can be grouped into three main families, namely, ERK, p38, and JNK/MAPKs (48). Activation of these three MAPKs has been linked to the induction of PTGS2 expression in various cell types (35, 50–52). However, in amnion fibroblasts, only activation of ERK and p38 but not JNK was found to be involved in the induction of PTGS2 expression by SAA1 with NFκB as a possible downstream effector (35). Because there exists an NFκB binding site in the promoter of *PTGS2*, we postulate that NFκB may be a transcription factor downstream to the P38/ERK1/2 pathway in the induction of *PTGS2* expression by BK and DABK. In this study, we also found that JNK was not implicated in the induction of PTGS2 by both BK and DABK despite that JNK was phosphorylated. The exact role of JNK activation by BK and DABK in amnion fibroblasts awaits further study.

Bacterial infection is the most common cause of preterm birth. LPS is a major component of the outer membrane of gram-negative bacteria, which plays a key role in host–pathogen interactions in the immune system. Activation of TLR4 by LPS induces the expression of critical proinflammatory factors including cytokines, PTGS2, and SAA1 in the fetal membranes (25, 35). Although SAA1 is known to be released primarily from the liver in the acute phase of infection, emerging evidence indicates that non-hepatic tissues including the placenta and fetal membranes are also capable of *de-novo* synthesis of SAA1 (35, 53). Like LPS, SAA1 has also been shown to interact with TLR4 to induce PTGS2 expression in amnion fibroblasts (35). Here, we demonstrated that both LPS and SAA1 induced the expression of BDKRB1 and BDKRB2 through TLR4 in amnion fibroblasts, which potentiated the induction of PTGS2 expression by BK and DABK. These findings provided a novel bradykinin-mediated mechanism of infection-induced preterm birth.

In conclusion, this study provides evidence that the human amnion is the target tissue of bradykinin peptides but not their source tissue. The bradykinin system in human amnion may be involved in both term and preterm birth with or without infection through, at least in part, stimulation of PTGS2 expression and subsequent PGE2 production in amnion fibroblasts.

## Data Availability Statement

The datasets presented in this study can be found in online repositories. The names of the repository/repositories and accession number(s) can be found below: https://www.ncbi.nlm.nih.gov/, GSE166453.

## Ethics Statement

The studies involving human participants were reviewed and approved by the Ethics Committee of Ren Ji Hospital, School of Medicine, Shanghai Jiao Tong University. The patients/participants provided their written informed consent to participate in this study.

## Author Contributions

X-TN, W-SW, ML, and KS designed the study, analyzed the data, and wrote the manuscript. X-TN, Y-KL, FZ, W-JL, L-JL, FP, Y-NZ, and M-DL performed the experiments. X-TN, YL, TD, and ML collected and analyzed the samples from the patients. All authors contributed to the article and approved the submission.

## Funding

This study was supported by the National Natural Science Foundation of China (81830042 and 82071677) and Shanghai Municipal Health Commission (2019SY044).

## Conflict of Interest

The authors declare that the research was conducted in the absence of any commercial or financial relationships that could be construed as a potential conflict of interest.

## Publisher’s Note

All claims expressed in this article are solely those of the authors and do not necessarily represent those of their affiliated organizations, or those of the publisher, the editors and the reviewers. Any product that may be evaluated in this article, or claim that may be made by its manufacturer, is not guaranteed or endorsed by the publisher.
